# Cancer

**Published:** 1876-03

**Authors:** E. H. Gibbs


### *CANCER.*

BY E. H. GIBBS, A.M., M.D.

It is not long since physicians regarded *true* cancer as incurable. When cases of cure were reported, as they were occasionally, it was claimed that they were not cases of real cancer.

We were among the number of those who had no faith in remedies, and who believed the disease necessarily fatal. We had a contempt for a class of men known as cancer-doctors, and never doubted but that their pretended cures were deceptive; that the so-called cancer was, usually, a scrofulous tumor or ulceration, or some non-malignant sore. But a positive diagnosis of these affections is now easily made, and it is found, and we believe admitted by most physicians who have investigated the subject carefully, that almost every case of cancer may be radically cured before it has commenced to ulcerate ; and that after the ulcerative process has commenced, the disease soon becomes constitutional on account of the absorption of the poisonous matter into the system; thus forming a cancerous diathesis which no remedies that have yet been discovered will eradicate from the blood. Still the disease is not necessarily fatal even when it has reached this stage. It may appear at intervals of three or four years in a new spot, aud then must be eradicated as at first.

In the treatment of cancer in its various stages, we most heartily approve of the plan adopted by Dr. A. H. Brown, whose success has certainly surprised his medical friends. He never uses the knife, believing that it invariably aggravates the disease ; but he removes the skin by means of blisters, and then applies an apparently mild dressing directly upon the diseased growth. The application attacks the diseased portion, but seems to have no effect upon the sound flesh beneath it. The preparation is left on from one to five days, depending on the size of the tumor; then a poultice of flax seed is applied, and this very soon causes a complete separation of the cancer from the healthy portion beneath it. Not a particle of the malignant growth is allowed to remain. The opening is then healed and the cure is complete.

